# Human Rights and Empowerment in Aged Care: Restraint, Consent and Dying with Dignity

**DOI:** 10.3390/ijerph18157899

**Published:** 2021-07-26

**Authors:** Tiffany Jessop, Carmelle Peisah

**Affiliations:** 1 Capacity Australia, P.O. Box 6282, Kensington, NSW 1466, Australia; cpeisah62@bigpond.com; 2 School of Psychiatry, UNSW Sydney, Sydney, NSW 2052, Australia

**Keywords:** human rights, chemical restraint, consent, empowerment

## Abstract

The aged care system in Australia is in crisis and people living with dementia are especially vulnerable to breaches of human rights to autonomy, dignity, respect, and equitable access to the highest quality of health care including meeting needs on account of disability. To be powerful advocates for themselves and others, people with dementia and the wider community with vested interests in quality aged care must be informed about their rights and what should be expected from the system. Prior to the Australian Royal Commission into Aged Care Quality and Safety, the Empowered Project was established to empower and raise awareness amongst people with dementia and their families about changed behaviours, chemical restraint, consent, end of life care, and security of tenure. A primary care-embedded health media campaign and national seminar tour were undertaken to meet the project aims of awareness-raising and empowerment, based on 10 Essential Facts about changed behaviours and rights for people with dementia, established as part of the project. Knowledge translation was assessed to examine the need and potential benefit of such seminars. We demonstrated that this brief educational engagement improved community knowledge of these issues and provided attendees with the information and confidence to question the nature and quality of care provision. With the completion of the Royal Commission and corresponding recommendations with government, we believe the community is ready to be an active player in reframing Australia’s aged care system with a human rights approach.

## 1. Introduction

In theory, full and equal enjoyment of human rights is owed to all. In practice, these rights are often not actualized by older people, particularly those living with dementia. A “Human Rights-based approach to aged care” has become a tag line from the recent Australian Royal Commission into Aged Care Quality and Safety (The Royal Commission) [1], although this, however, is not a new concept. The structures around, and the delivery of aged, or elder care are fraught with contraventions of human rights of older people, prompting debate for over a decade about the need for an international convention on the rights of older persons [2]. As implied by the term ‘international’, this problem is global, and the COVID-19 pandemic has brought these inequalities, discrimination, and stigma front and center [3]. The International Psychogeriatric Association (IPA) and the World Psychiatric Association Section of Old Age Psychiatry (WPA-SOAP) suggest as a remedy for this huge shortfall in rights-based care and service provision, involvement and consultation with ‘consumers’ of this care, the people with lived experience [3].

The over-use of restraint in aged care is a prime example of human rights violations of people with disabilities in regards to (i) autonomy; (ii) dignity; (iii) equitable access to the highest attainable standard of health care, including meeting needs on account of disability; and (iv) safeguarding against abuse and torture and cruel, inhuman, or degrading treatment or punishment [3,4]. These rights are articulated in the Convention on the Rights of People with Disabilities [5].

Prime amongst these is a failure to respect autonomy, manifested by failure to obtain consent (including substitute consent where appropriate) prior to the administration of chemical restraint. Again, this is longstanding [6,7,8]. Notably, there is both anecdotal and research evidence that family members who are designated or default decision makers are neither consulted in regard to consent for chemical restraint [8], nor informed about major side effects including mortality risk, while often subject to pressure by Residential Aged Care Facilities (RACFs) to accede to such restraint [8,9] for fear of the resident with Changed Behaviours losing tenure in the facility. In a recent study of antipsychotic deprescribing (reducing medication) to which members of our research team contributed, Halting Antipsychotic use in Long-Term care (HALT), substitute decision makers were interviewed prior to deprescribing and in the event that an antipsychotic was re-prescribed after a period of withdrawal [10]. These interviews revealed that in many cases family members were neither informed of prescribed psychotropics, nor provided with any information regarding risks and benefits of such. Responses demonstrated the fear and helplessness faced by families when they lack information and knowledge and rely solely on medical professionals to make the “right” decision when acting as a substitute decision maker [9]. Comments such as “Leave it to the staff and GP to decide what’s needed” and “… long time to find the right home for him, not risking him being kicked out” were from family members who conceded there may be a need for medications; while there were also family members who were firmly against antipsychotic prescribing but felt powerless to stop it “Not happy, would rather she yell at people…” [10].

The Australian Commission for Safety and Quality in Health Care’s Roundtable on Reducing inappropriate use of antipsychotics for older people (October 2016) stressed the importance of involving consumers (person with dementia and their ‘carer’) in bringing about change with this issue. This is consistent with their overarching NSQHS Standard 2 Partnering with Consumers, and in the context of their Standard on Medication safety. Psychotropic use is but one focus for partnering with and empowering consumers to exercise choice and control in the planning and delivery of aged care, as recommended by the Royal Commission [1]. To empower is to impart strength and confidence, especially in regard to claiming rights. WHO defines empowerment as “a process through which people gain greater control over decisions and actions affecting their health” [11], and this notion is endorsed by the United Nations with empowerment listed as one of the five key principals for a rights-based approach for people with dementia [12].

Older people, people living with dementia, and their families and supporters have the right to meaningfully participate not only in personal health-care decision making such as medication use, but also in other prime areas pertinent to their human rights including the right to a good death [13,14], security of tenure [15], equity issues in relation to LGBTQ+ elders [16], and the harms associated with hospital admission for people with dementia [17,18]. They can only do so if they are informed. Previous studies, particularly regarding quality use of psychotropics, have focused on the aged care and clinical workforce [18,19,20,21]. Shelton et al. [22] refer to the “Dementia Care Triad” as the partnership between the person with dementia, family carers, and healthcare team, each being an essential component. Our previous research and clinical experience led us to identify a clear gap in the approach to quality care for people experiencing changed behaviours and carers being left “out of the loop”, substantially diminishing the potential benefit of interventions with a purely staff or care-provider focus.

Our team established the Empowered project (Empowering people with dementia and their proxy decision makers regarding deprescribing) with funding from the Australian Commonwealth Department of Health Dementia Aged Care Service Grant, to empower this target group, people living with dementia and their carers, with information related to restraint use and beyond. Our previous work in both the aged care and human rights fields alerted us to a distinct lack of targeted information for a lay audience (i.e., community, people with dementia and their families) about changed behaviours, psychotropic medicines, non-pharmacological alternatives, and more general rights of people living with dementia residing in aged care facilities. The project aimed to address this gap in approaches to practice change in this area and target this information to people living with dementia and their carers, who play a critical role in treatment and care decisions when the person with dementia no longer has capacity to make these decisions alone.

The Empowered project aimed to educate and raise awareness amongst people living with dementia and their families, with information about changed behaviours, restraint use, and their rights in relation to aged care. We hypothesised that (i) awareness-raising would occur following a multi-media education campaign integrated into GP surgeries; and (ii) knowledge translation would occur following delivery of face-to-face community seminars.

## 2. Methodology

**Figure 1:**
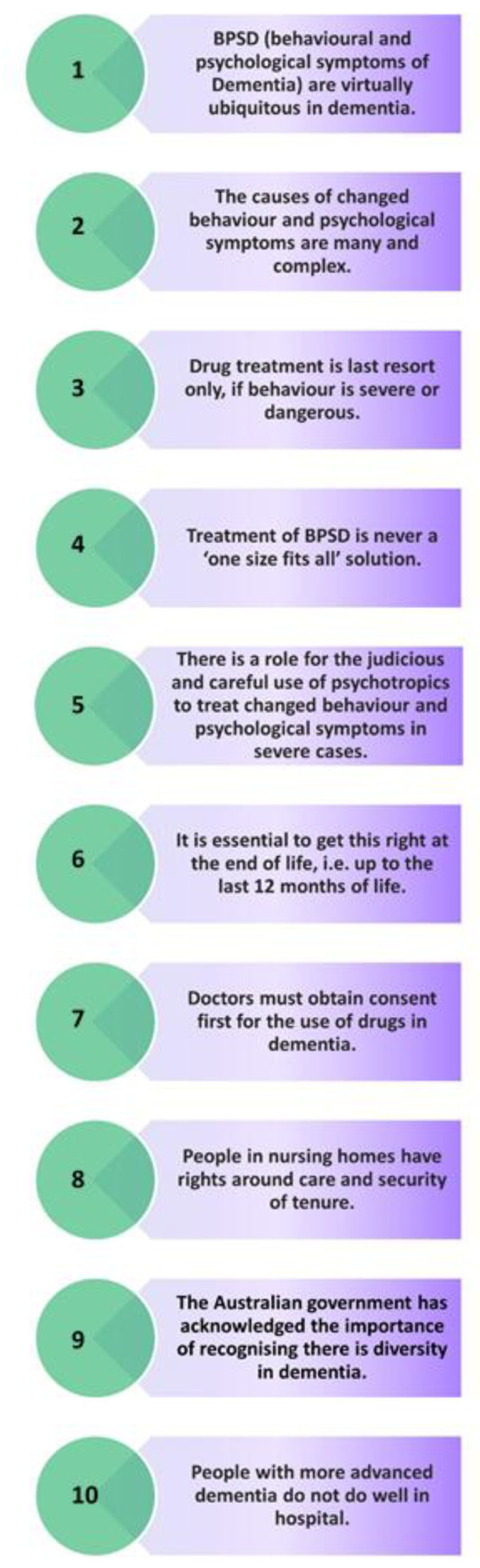
Empowered 10 Essential Facts regarding changed behaviours and rights in dementia—Clinican Version.

A review of the recent literature was undertaken guided by expert input from the research team’s advisory group, to establish the core messaging of the project and the main focus points for awareness-raising about changed behaviours in dementia, chemical restraint, and rights in relation to aged care (see Supplementary Materials for a list of references). Sources included original research, systematic reviews and meta-analyses, clinical guidelines, and grey literature. The main points of note from our experts and the literature included information about medications, consumer rights, and quality care, and were called the “10 Essential Facts” (Figure 1). These Essential Facts were named as such based on the recent experience of the research team (clinical practice and the HALT project mentioned above) and identified priorities for practicing change relating to changed behaviours. Three approaches were taken to raise awareness of the 10 Essential Facts in the community: (1) a national community education and awareness campaign utilising an experienced health media company; (2) a face-to-face seminar program delivered to community members, people with dementia, and carers incorporating a knowledge translation evaluation; and (3) a dedicated website to act as a repository of resources.

(i) The national awareness-raising intervention incorporated a 60 s TV Commercial (TVC) supported by a print brochure campaign and digital panels present in over 1500 GP practices, over a 3-month period. The evaluation of this aimed to measure:

- patient engagement with the campaign, including whether they listened to the TVC, picked up a brochure, or noticed a digital panel.
- Any action resulting from engagement including visiting the Empowered website, talking about it with their GP, or passing information on to a friend.
- Recall of the Empowered Project campaign amongst patients, including awareness and key messages.
- Attitudes towards the Empowered Project campaign amongst patients, including relevance and intention to act.

A quantitative, in-practice evaluation was conducted to assess the effectiveness of the campaign and capture if consumers engaged with the content, and what action was taken. A team of three representatives from the Health Media company conducted the face-to-face surveys in practice waiting rooms. The Survey Monkey platform was utilised so that the participants could complete the survey on iPads provided by the representatives while waiting to see their GP. Medical Practices with a high flow of patients (four or more full time GP’s) were used to conduct the interviews with data collected during practice opening hours (9 a.m.–5 p.m.). The surveys took place in metro and regional practices in NSW, QLD, and VIC to ensure an accurate representation of the entire population. Random sampling was undertaken from males and females approximately 40 years and over; however, the campaign is potentially relevant to all patients over 18 years.

Data was analysed for trends and insights by an independent consulting agency.

(ii) A face-to-face national seminar program was conducted over 1 year from July 2018 to August 2019. Participants were recruited to attend the seminars using a variety of means such as social media, mailouts via the research teams networks, and engaging dementia and aged care community organisations to in-turn promote the events to their clients. It was important for the project to reach as many interested Australians as possible, and seminars were held in both metropolitan and rural locations. In addition, to address a potential barrier of being unable to attend a physical seminar, a webinar was held and recorded for on-demand access via the project website. The face-to-face seminar included a 20-min presentation outlining each of the 10 Essential Facts and provided up to 30 min of discussion and questions from participants. Accompanying this verbal presentation, each seminar attendee was provided with a printed ‘brochure’ outlining the 10 Essential Facts and a list of useful contacts that may be beneficial for further information and support (all Empowered resources including this brochure can be found on the Empowered website www.empoweredproject.org.au, accessed on 22 July 2021).

To evaluate the impact of the seminars, a pre/post questionnaire was established to capture participant experience and knowledge change as a result of brief education. Ethics approval for this component of the study was granted by the UNSW Human Research Ethics Committee (HC17955). A Participant Information Statement and Consent Forms was provided to all seminar attendees on arrival, and they were given time to consider whether they would like to complete the pre/post questionnaires and ask questions about the evaluation. Attendees agreeing to participate in the evaluation signed the PISC and completed the “pre” knowledge questionnaire before the seminar and the “post” questionnaire after the seminar.

Data analysis was performed using IBM SPSS Statistics 23 (Armonk, NY: IBM Corp). Data collected from the pre/post surveys was paired where possible, however, a number of participants only completed either the pre or post questionnaires and not both. Frequency analysis was performed for all cases, while paired samples t-tests were conducted for paired cases only. Results were compared between those who identified as healthcare workers and those who did not.

## 3. Results

Following an extensive literature review, the Empowered 10 Essential Facts relating to changed behaviours in dementia and related rights in aged care were developed in both community and clinician versions (Table 1). These documents were the foundation for all key outputs and materials developed during the project (www.empoweredproject.org.au, accessed on 22 July 2021).

### 3.1. Health Media Campaign

The national health media campaign was successfully delivered over 3 months (April–June 2018) to waiting rooms in over 1500 GP surgeries around Australia. In addition, information about the project was delivered direct to 2654 GPs across Australia via email. The health media campaign resulted in higher than usual engagement from the public amongst those surveyed as well as amongst Australian GPs.

The evaluation survey conducted by health media representatives engaged with 251 consumers attending general practices at three of the GP sites across Australia: 66% of survey participants were female and 34% were male. Over 50% of participants were aged over 50 years.

The survey found that more than half (54%) of patients thought the topic (dementia) was relevant to them and 30% mentioned the topic of the brochure or the actual Empowered phrase unprompted. When shown the brochure and asked if they would use it, almost 80% said they would: more than two thirds (68%) said they would take it away and read it later, and more than 1 in 10 (11%) said they would give it to someone they knew who would find the information useful. Over half of the patients surveyed had watched TV in the waiting room at their appointment and of those who watched, one in five mentioned that they saw the content on dementia/changed behaviour or mentioned the actual Empowered phrase unprompted. When prompted whether they saw the advertising, 44% said they had seen the commercial; those who saw it said it made them concerned and interested; and over one third (39%) of those who were exposed to information on the project said they would go to the website for more information. Based on figures provided by the health media company, the digital campaign potentially reached >9 million people nationally over the 3 months and approximately 4 million people were exposed to the print component.

Information about the Empowered project was emailed to 2654 GPs across Australia. Electronic monitoring of email receipt/opening was enabled so that the senders (Health Media Company) could track who opened and took action regarding the email content. Over one quarter of GPs (26.5%) opened the Empowered direct message email (4% higher than the industry average) and 17.3% clicked through to read more information about the Empowered project, watch a GP-specific video presented by Dr Norman Swan, and view the 10 Essential Facts (clinician version, Figure 1). This result is approximately five times higher than the industry standard average click-through rate.

### 3.2. Face-To-Face Seminars

For the 12-month period July 2018 to August 2019, 28 face-to-face seminars were presented in six states of Australia across a range of urban and rural-regional locations. A multi- method approach to promotion, engagement, and attendance was utilised.

There were 165 participants in the Knowledge Exchange evaluation, a number (56) of these did not complete both pre and post-questionnaires and therefore could not be included in the paired analysis. Available demographic data are presented in Table 2. The majority of seminar participants were female. Over 50% of attendees were in the 46–70 years age bracket and there was equal representation across major cities, rural and regional areas, which fulfilled our aim of representation.

The pre-post knowledge questionnaires contained 12 items based on the content covered in the seminar presentation. We examined the change scores for all participants and split the data into those who identified as a “healthcare worker” and those who did not. This was based on the assumption that those who work in health and aged care may have higher baseline knowledge than the lay community, which would bias results. When looking at change in knowledge of the seminar content before and after the seminars, overall there was a significant improvement in participants’ knowledge (*p* < 0.000). When the change in scores was examined separately within the healthcare and the lay community groups, we found an increase in knowledge in both groups. However, while the change in scores (knowledge exchange) in the community group was significant (*p* < 0.02); there was only a non-significant trend for increase in healthcare knowledge scores (Table 3). This would be explained by our hypothesised higher levels of baseline healthcare worker knowledge and ceiling effect.

We then examined the frequency of correct, incorrect and “I don’t know” responses on a question-by-question basis for all cases (N = 165), paired and unpaired, to explore trends of consistently incorrect items or those unaddressed by the seminars. This also allowed us to look for future targets of education.

Table 4 provides a detailed look at the correct, incorrect and “I don’t know” responses for all 12 questions pre and post seminar. A positive outcome of the seminar was the vast reduction in participants responding with an “I don’t know” to questions. While for some questions the proportion of incorrect answers was unchanged between pre and post, the amount of uncertainty was reduced, thereby retaining the benefits of the knowledge exchange. Interestingly, Question 8: “The best way to address changed behaviours and psychological symptoms in dementia is to:” was almost universally answered correctly and rarely responded to with an “I don’t know” answer. The answer to this question (Find out why the behaviours are happening) aligns with the prevailing understanding of behaviours as communication of unmet needs, at the heart of person-centred care for people with dementia. In our sample of both health care workers and lay community members we find knowledge of this, and yet it is something that many care facilities are not able to implement in day-to-day care. This demonstrates that it is not lack of community or professional knowledge that prevents person-centred approaches to Changed Behaviours, or advocacy for such, but perhaps other more practical barriers such as resourcing.

Conversely, there was relatively poor understanding of the role of pain and its treatment in Changed Behaviours, and this was highlighted by the incorrect or ‘I don’t know” responses to Question 11 (collectively almost 60% of responses). Similarly, defining the end of life in dementia and initiating comfort care was an important knowledge gap that responded to the campaign, as did understanding of the potentially harmful environments hospitals pose for people with dementia. These areas remain important targets for future education.

### 3.3. Consumer Feedback

At the completion of the post questionnaires, participants were provided with the opportunity to comment further about the seminar or their lived experiences that may have brought them to the seminar. Overwhelmingly, these comments reflected the usefulness of the seminars, reinforcing the need for such information to be available to healthcare and community alike.

“There needs to be more public education/awareness like this seminar, to improve awareness in the community about this important issue, which has such wide-ranging and severe impacts on people’s lives and their families.”

“I found this very empowering and have a list of questions now to ask my father’s care providers”

While information-sharing was the initial thrust of the Empowered message, encouraging question-asking of health care providers subsequently became the dominant message. In this way, the Empowered campaign became somewhat of an iterative process, responding to the needs of attendees. Notably, much of the Empowered campaign overlapped with the themes that emerged from the Royal Commission and much of the tenor of the feedback reflected the inadequacy of care and understanding of older people, and the frustrations of carers:

“Attitudes from care staff are not always focused on client’s rights to choose, be informed and make decisions. People see the word dementia and equate it to someone who can’t make decisions or be informed “

“My grandfather has dementia for about 5–10 years …He was often sad, depressed or angry… He was badly treated by some hospital and nursing home staff, who locked him in a room and neglected him.”

## 4. Discussion

There is a need for community awareness about upholding basic human rights in aged care as well as rights to quality care, particularly for people with dementia and those with changed behaviours. This was apparent prior to the Empowered project and was since reinforced during the proceedings of the Royal Commission into Aged Care Quality and Safety [1]. To the best of our knowledge, the Empowered Project was the first of its kind to specifically target consumers with education and awareness-raising resources related to care for people living with dementia, with the aim of empowerment and increased engagement in decision making. This gap was clearly evidenced by the recent Royal Commission’s Recommendation 26: “Improved Public Awareness of Aged Care”, which has supported similar initiatives as part of the Aged Care Reform [1]. The Empowered project was successful in reaching a modest number of engaged Australians and perhaps off the back of the Royal Commission, the public will be more willing players in this endeavour.

Alongside the Royal Commission, the Australian Government has established an Aged Care Clinical Advisory Committee (ACCAC) specifically looking at Reducing the Inappropriate use of Restraint in Residential Aged Care. Moreover, neither use of restrictive practices in aged care nor obligations of RACFs under the Aged Care Act nor specific use of best practice person-centred care is made transparent to prospective users of facilities. RACFs are not graded on these critical points of care or given incentives for demonstrated best practice beyond the tick box approach required to meet accreditation standards. It is hoped that the activities of the ACCAC and impact of the Royal Commission will lead to changes in policy and procedures around the “tick box” approach for accreditation purposes for restraint use but more generally also.

The Empowered project team made a number of recommendations to the sponsor at the conclusion of the project. This was delivered prior to the Royal Commission findings and many of these recommendations have been subsumed in the extensive list of recommendations of the Commission. These included dementia-specific training for health and age care staff, particularly around changed behaviours; more tailored resources for people living with dementia and their families regarding psychotropic medicines and complimentary information for aged care staff; changes to the fee structure for prescribers so that they are duly compensated for the time needed to adequately care for patients living in residential aged care. Prime amongst recommendations is the inclusion of practice change and policy development around consent for psychotropic use, including revision of the Quality of Care Amendment (Minimising the Use of Restraints) Principles 2019 [23] and making use of the new electronic prescribing system to embed documentation of consent at the time of prescribing [6]. In addition, one of the significant outcomes of the Royal Commission is the final marrying of aged care with human rights and a commitment from policy makers to approach reform of the Aged Care act through a human rights lens with dignity, respect, and preferences front of mind [1].

Additionally, we identified other areas for future targeted community education. Notably, more than half of the cohort attending the face-to-face seminars under-estimated the role of pain driving changed behaviours and the role of analgesia, despite its known role in relieving agitation [24]. This may partly explain under-medication of pain amongst people with dementia [25].

End of life and the provision of comfort care are also important targets for consumer empowerment. The Empowered seminars resulted in a 50% increase in correct answers to this item and a 12-fold reduction in “I don’t know” responses. To this end, a user-friendly brief video was developed to enhance early initiations of such discussions and address fears regarding referral to palliative care [26]. Clearly, consumer and carer partnerships are integral to quality dying [27].

When consumers are informed, they know what to look for, but performance of aged care providers must be signposted widely, beyond regulators and government agencies. Community knowledge translation must be augmented with greater transparency from providers with an accountability framework, for which we have been long-term advocates.

Of course, Australia is not alone in its challenge to improve care for older people, and as previously mentioned, there are international initiatives to advocate for a rights-based approach to aged care (UN, IPA). Australia’s pressing need for reform and consumer- driven improvement in aged care is motivated by our relatively low financial investment in the aged care sector. A review of aged care provision in 13 countries found that Australia has one of the highest proportions of older people receiving long-term aged care but contributes the smallest percentage of their GDP to aged care service provision [28]. Addressing this funding lag is a logical first step for change.

In its most recent budget release, the Australian Government has allocated $17.7 billion over 5 years to address the shortcomings of the current aged care system, including the recommendations from the Royal Commission [29]. This includes $200.1 million to introduce a new star rating system to provide senior Australians, their families, and carers with information to make comparisons on quality and safety performance of aged care providers; $74.8 million for the Dementia Behaviour Management Advisory Service and the Severe Behaviour Response Teams to strengthen the regulation of chemical and physical restraints, and to further reduce reliance on these restraints; and $216.7 million over 3 years from 2021–2022 to grow and upskill the workforce and enhance nurse leadership and clinical skills including dementia and palliative care [29].

## 5. Limitations

Potential barriers to community engagement with the face-to-face program were explored and adaptive solutions crafted such as exposure at large existing community events and developing several webinar seminars and website resources. Furthermore, the cohort of people who turned up to the face-to-face seminars were likely to be more educated about these issues and unlikely to be reflective of the wider population of consumers. It is possible that an added benefit of the Royal Commission is that it has brought these issues to the broader community and they are now primed for action.

## 6. Conclusions

Partnerships between research, industry, and policy will ensure that resources developed by, and outcomes from community awareness-raising projects such as this, have sustainable impacts. Success of awareness-raising, empowerment, and practice change initiatives rely on timing and readiness for change. The Empowered project preceded the Royal Commission by 1 year, but now is the time to strike with change.

## Figures and Tables

**Table 1:** Empowered 10 Essential Facts regarding Changed Behaviours and rights in dementia care.

Essential Fact Number	Consumer/Carer Version By-Line	Consumer/Carer Version (Detailed VERSION)	Medical Version (Doctors and Nurses) By-Line	Medical Version (Doctors and Nurses) Detailed Version
1.	Behaviour changes and psychological symptoms are as common in dementia as memory loss.	Dementia is frequently associated with psychological symptoms and changes in behaviour. The person with dementia might say or do things that they wouldn’t have previously. Up to 90% of people with dementia experience this changed behaviour or psychological symptoms. These are known as BPSD (behavioural and psychological symptoms of dementia)	BPSD (behavioural and psychological symptoms of dementia) are virtually ubiquitous in dementia.	BPSD are behavioural and psychological symptoms of dementia. They effect up to 90% of people with dementia.
2.	Psychological symptoms and behaviour change with dementia have a number of causes.	Changed behaviour and psychological symptoms in people with dementia have a number of causes. It may be caused by changes in the brain associated with dementia; the person may be unwell (e.g., have an infection); or commonly the person has an unmet need that they cannot express such as pain, temperature, hunger, thirst, boredom, need for nurturing/intimacy or loneliness. Sometimes the person is having trouble understanding or making sense of their environment.	The causes of BPSD are complex	The causes of BPSD are complex and include a range of biopsychosocial and environmental causes including: (I) acute medical illness or frank delirium; (II) unmet needs such as pain, nurturance and intimacy, hunger, temperature discomfort, and loneliness, which may not be able to be expressed or understood by the person; (III) a response to, or an attempt to make sense of, the environment, such as fear or confusion; (IV) the disease process itself—structural and biochemical neurotransmitter changes occurring in the brain during the course of dementia can lead to symptoms such as irritability, screaming, delusions, andhallucinations.
3.	Non-drug treatment first—Drugs are a last resort unless the behaviour is severe.	The approach to prevent or minimise distress for the person with changed behaviour and psychological symptoms should always be without medicines first, unless the behaviour poses a risk to the person or those around them, or likely to respond to medicines (e.g., paranoia).	Drug treatment is last resort only, or if BPSD are severe.	Treatment is always non-pharmacological (non-drug) first unless behavior is associated with significant risk/distress to the patient or others, or likely to respond to psychotropics. There is a place for positive prescribing in dementia: eg cholinesterase inhibitors and antipsychotics for paranoid symptoms and for severe agitation/aggression.
4.	The best way to address changed behaviours and psychological symptoms in dementia is to find out why they are happening.	The “trick” to the non-drug approach to changed behaviours and psychological symptoms is to work out the biological, psychological or social/environmental cause including unmet needs. Is the person unwell? Do they need something and can’t express it? Are they in pain, frightened or lonely? This is very individual and may also be influenced by personal life experiences and is thus called “person-centred care”. No “one size fits all” solution will work. There is scientific evidence that many of these non-drug solutions work, including music therapy and training staff in communication and understanding the individual needs of people with dementia. Dementia Support Australia (DSA) is a national service, which administers the Dementia Behaviour Management Advisory Service (DBMAS) and Severe Behaviour Response Teams (SBRT) to up-skill, assist, and support aged care providers in improving care for people with dementia and related behaviours that are responsive to their individual and diverse needs and circumstances.	Treatment of BPSD is never a ‘one size fits all’ solution.	Treatment strategies are never a ‘one size fits all’ solution, necessitating an individualised person-centred approach to BPSD, which requires a comprehensive assessment of the causes including personal history. There is a growing evidence base for the efficacy of a range of psychosocial/environmental strategies, focused on “person-centred care”. Evidence exists for music therapy, home-based behavioural management techniques, caregiver-based interventions or staff training in communication skills, and person-centred care or dementia care mapping with supervision during implementation. Often recourse to drugs occurs when the professional care environment is insufficiently resourced or informed/educated. Dementia Support Australia (DSA) is a national service, which administers the Dementia Behaviour Management Advisory Service (DBMAS) and Severe Behaviour Response Teams (SBRT) to up-skill, assist, and support aged care providers in improving care for people with dementia and related behaviours that re responsive to their individual and diverse needs and circumstances. Their 24 h helpline number is 1800 699 799.
5.	“Never say never” about drugs in dementia: Sometimes medicines can be helpful but they have a number of side effects, some serious.	Only after non-medicine approaches have failed, if the person is very distressed or the behaviour is severe or dangerous should medication be considered. Paranoid delusions may cause distress and respond favourably to medications. Some medicines used to help and psychological symptoms and changed behaviours have serious risks associated with them such as sedation, falls, stroke, and death. Medication should be started at the lowest dose, response and side effects should be monitored closely, and the dose of medicine adjusted accordingly, and the person should be reviewed at least every 3 months to see if they still need medicines. Anti-dementia drugs (which are misnamed because they don’t stop dementia) also have a role in improving cognition and function. Simple list of psychotropics for consumers.	There is a role for the judicious and careful use of psychotropics to treat BPSD in severe cases.	Psychotropic drugs should only be used for the treatment of agitation/aggression or psychosis in patients with dementia when symptoms are severe, dangerous, and/or cause significant distress to the patient. Certain psychotic symptoms such as paranoid delusions may cause distress and respond favourably to medications. If medications are indicated, they should be used for the shortest time at the lowest possible dose and be reviewed at least every 3 months. Psychotropic drugs have side effects and are associated with stroke and death plus sedation, falls, QT prolongation, pneumonia, and extrapyramidal side effects. Cholinesterase inhibitors or “anti-dementia drugs” also have a role in in improving cognition and function in dementia.
6.	If ever there was a time to understand changed behaviours, psychological symptoms, or unmet needs, it is at the end of life, including the last 12 months of life, when quality of life is a priority.	It is essential to get this right at the end of life. This is perhaps the most important time to understand and address unmet needs such as pain, anxiety, fear, and loneliness or physical causes of distress. Medications aren’t effective in treating delirium at the end of life. It is also important to focus on the person’s wishes and priorities in regards to end of life care.	It is essential to get this right at the end of life (not just the last few hours of life).	It is essential to get this right at the end of life (not just the last few hours of life). Human rights of equitable access to health care, the relief of distress and pain, and to autonomous decision making are at stake. In patients receiving palliative care, individualized management of delirium precipitants and supportive strategies are more efficacious in treating distressing delirium symptoms than risperidone or haloperidol.
7.	Consent before use: Doctors must obtain consent first for the use of drugs in dementia.	All doctors (including GPs and specialists) are required to obtain consent from the person themselves where possible, or if they cannot give consent, from their proxy or substitute decision maker, often called the person responsible, for prescribing medicines used to help changed behaviour. This consent needs to be informed i.e., the material risks and benefits of the drug need to be explained. The only exception is in an emergency and then consent must be sought as soon as possible after administration.	Doctors must obtain consent first for the use of drugs in dementia.	If drugs are going to be used, they need to be given with consent, except in an emergency. Clinicians have a duty to ensure that patients are aware of any material risks involved in a proposed treatment and of reasonable alternatives to that treatment. The absence of a valid consent is a factor in establishing liability for civil assault or trespass. For medical professionals, criminal responsibility could arise for murder or manslaughter (where treatment is withheld or withdrawn unlawfully) or for assault (where treatment is provided without appropriate consent or authorisation). Human rights of equitable access to health care, the relief of distress and pain, and to autonomous decision making are at stake.
8.	People in nursing homes have rights around care and security of tenure.	4. People in nursing homes have rights around care and in their tenure. They can’t be “evicted” simply because of what others perceive as “bad behavior”. Compliance with the User Rights Principles 2014 (s 96-1 Aged Care Act 1997) Security of tenure is compromised when facilities transfer patients to hospital due to “unmanageable” BPSD, with threat of loss of a bed. A person cannot be asked precipitously to leave a nursing home without prior specialised and independent assessment, written notice, and available alternative accommodation.	People in nursing homes have rights around care and in their tenure.	People in nursing homes have rights around care and in their tenure. Compliance with the User Rights Principles 2014 (s 96-1 Aged Care Act 1997) Security of tenure, is compromised when facilities transfer patients to hospital due to “unmanageable” BPSD, with threat of loss of a bed. A person cannot be asked precipitously to leave a nursing home without prior specialised and independent assessment, written notice, and available alternative accommodation. Dementia Support Australia (DSA), Severe Behaviour Response Teams (SBRT) provide a 24 h helpline number—1800 699 799.
9.	Special needs groups deserve special attention.	The Australian government has acknowledged the importance of recognising the needs and rights of special needs groups such as Lesbian, Gay, Transgender, Bisexual and Intersex communities, Culturally and Linguistically diverse communities, and Aboriginal and Torres Strait Islander communities in aged care.	The Australian government has acknowledged the importance of recognising the needs and rights of special needs groups such as LGTBI, ATSI & CALD communities.	Always consider special needs groups such as LGTBI, ATSI & CALD communities. The Aged Care (Living Longer Living Better) Bill 2013 allowed for expansion of the meaning of ‘people with special needs’ under Section 11.3(h) of the Aged Care Act 1997 to include “lesbian, gay, bisexual, transgender and intersex people” under the subsequent Allocation Principles 2014 (Section 26(a) and 29). Notes also rights under Guardianship Act 1987. Also, the “same sex partner” (assuming they are recognised as such) has the same rights as any spouse to act as the Person Responsible or Statutory Health Attorney to give proxy treatment consent on behalf of a partner unable to give consent themselves.
10.	People with more advanced dementia do not do well in hospital.	People with more advanced dementia do not respond well to the hospital environment. The negative outcomes associated with hospitalization of people with dementia include fractures, head injuries, skin tears, infections, inappropriate sedation, and death.	Negative outcomes are associated with hospitalization of people with dementia.	There are a range of negative outcomes associated with hospitalization of people with dementia including fractures, head injuries, skin tears, infections, inappropriate sedation, and death.

**Table 2:** Demographics of the participants in the Empowered Knowledge Translation evaluation.

Item	Categories	N (%)
Gender	Male	26 (18.7)
	Female	112 (80.6)
	Prefer not to say	1 (0.7)
Age	18–30	11 (7.9)
	31–45	18 (12.9)
	46–60	45 (32.4)
	61–70	33 (23.7)
	71–80	18 (12.9)
	81–90	13 (9.4)
	Prefer not to say	1 (0.7)
State	ACT	3 (2.2)
	NSW	54 (38.8)
	NT	1 (0.7)
	QLD	46 (33.1)
	SA	17 (12.2)
	TAS	13 (9.4)
	VIC	3 (2.2)
	WA	2 (1.4)
Metro Type	Major City	37 (27.4)
	Surburban	38 (28.1)
	Regional	32 (23.7)
	Rural	28 (20.7)
Employment	Full-time	44 (31.7)
	Part-time/Casual	31 (22.3)
	Unemployed, looking for work	1 (0.7)
	Unemployed, not looking for work	1 (0.7)
	Retired	56 (40.3)
	Prefer not to respond	6 (4.3)
Work in Health or Aged care (n = 81)	Nurse	15 (18.5)
	Facility Manager	1 (1.2)
	Psychologist	3 (3.7)
	Geriatrician	2 (2.5)
	Aged Care Worker	13 (16)
	Allied Health	12 (14.8)
	Other	35 (43.2)

**Table 3:** Changes in overall scores between pre- and post-knowledge exchange assessments.

		PRE	POST
Health Worker	Mean	9.2667	10.8378
	N	45	37
	Std. Deviation	1.69759	1.04119
	Median	9.0000	11.0000
	Minimum	2.00	7.00
	Maximum	12.00	12.00
	Sig (2-tailed)	0.002	
Not Health Worker	Mean	6.6667	10.0517
	N	69	58
	Std. Deviation	3.20692	1.87712
	Median	7.0000	11.0000
	Minimum	0.00	4.00
	Maximum	12.00	12.00
	Sig (2-tailed)	0.000	
Total	Mean	7.6930	10.3579
	N	114	95
	Std. Deviation	2.99003	1.64327
	Median	9.0000	11.0000
	Minimum	0.00	4.00
	Maximum	12.00	12.00

**Table 4:** Pre/post results for all items on the Knowledge Exchange questionnaire.

	PRE			POST		
	Correct n (%)	Incorrect n (%)	Don’t Know n (%)	Correct n (%)	Incorrect n (%)	Don’t Know n (%)
Q1 Dementia is a normal part of ageing (True/False)	95 (57.6)	21 (12.7)	17 (10.3)	114 (69.1)	14 (8.5)	5 (3)
Q2 Changed behaviours and psychological symptoms occur in what % of people with dementia? * up to 3% * up to 20% * up to 60% * up to 90% * I don’t know	48 (29.1)	19 (11.5)	33 (20)	99 (60)	17 (10.3)	14 (8.5)
Q3 People with advanced dementia do well when they are in hospital (True/False)	89 (53.9)	5 (3)	38 (23)	116 (70.3)	7 (4.2)	9 (5.5)
Q4 In treating changed behaviours and psychological symptoms in dementia, which of the following is true: * Sedative medications are too risky and should never be used * Sedative medications should be used if in severe or high risk situations * Sedative medications should always be used, it’s the only way to manage changed behaviours * I don’t know	83 (50.3)	16 (9.7)	32 (19.4)	107 (64.8)	20 (12.1)	4 (2.4)
Q5 Nursing homes have no particular obligations towards Lesbian, Gay, Bisexual, Transgender or Intersex (LGBTI) elders (True/False)	75 (45.5)	20 (12.1)	38 (23)	107 (64.8)	17 (10.3)	7 (4.2)
Q6 There is NO service available to assist nursing homes support people experiencing changed behaviours or psychological symptoms in dementia (True/False)	88 (53.3)	4 (2.4)	40 (24.2)	114 (69.1)	4 (2.4)	11 (6.7)
Q7 If a doctor thinks that a person with dementia needs a sedative to calm them down, they don’t need to get informed consent from that person, or their carer to prescribe these medications (True/False)	87 (52.7)	13 (7.9)	32 (19.4)	118 (71.5)	10 (6.1)	4 (2.4)
Q8 The best way to address changed behaviours and psychological symptoms in dementia is to: * Sedate the person with medication to calm them down * Be firm with the person and explain to them that they are just being silly * Find out why the behaviours are happening * I don’t know	116 (70.3)	0	15 (9.1)	129 (78.2)	0	2 (1.2)
Q9 A person can be asked to leave, or be discharged from a nursing home if they: * behave in an aggressive manner or if they cannot be cared for * if they cannot be cared for and have specialised and independent assessment, written notice, and available alternative accommodation * I don’t know	76 (46.1)	14 (8.5)	42 (25.5)	123.(74.5)	2 (1.2)	6 (3.6)
Q10 Uncharacteristic behaviours in a person experiencing dementia are generally a response to unmet needs (True/False)	77 (46.7)	18 (10.9)	35 (21.2)	115 (69.7)	10 (6.1)	6 (3.6)
Q11 Pain relief and morphine-like drugs are never the answer for changed behaviours and psychological symptoms of dementia (True/False)	34 (20.6)	53 (32.1)	43 (26.1)	70 (42.2)	57 (34.5)	4 (2.4)
Q12 In dementia, acknowledging the end of life and focusing on comfort care should occur: * only in the last few hours of life * only in the last few days of life * up to one month before the end of life * up to 6–12 months before the end of life * I don’t know	81 (49.1)	8 (4.8)	41 (24.8)	122 (73.9)	3 (1.8)	4 (2.4)

## Supplementary Materials

The following are available online at https://www.mdpi.com/article/10.3390/ijerph18157899/s1, Empowered 10 Essential Facts reference list.
