# Supplementary material for: Human Rights and Empowerment in Aged Care: Restraint, Consent and Dying with Dignity
Source: Int J Environ Res Public Health. 2021 Jul 26;18(15):7899. doi: 10.3390/ijerph18157899 (PMC8345762; doi:10.3390/ijerph18157899)
Supplement: Supplementary file 1 [file ijerph-18-07899-s001.zip › ijerph-1279020-supplementary.pdf]

International Journal of
Environmental Research and Public Health

MDPI

Supplementary Material

Empowered 10 Essential Facts reference list

Aalten P, de Vugt ME, Jaspers N, et al. The course of neuropsychiatric symptoms in dementia. Part II: relationships among behavioural sub-syndromes and the influence of clinical variables. Int J Geriatr Psychiatry 2—5; 20: 531–36.

Abraha Rimland JM, Trotta FM, et al. Systematic review of systematic reviews of non-pharmacological interventions to treat behavioural disturbances in older patients with dementia. The SENATOR-OnTop series. BMJ Open 2017; 7: e012759.

Achterberg WP, Pieper MJ, van Dalen-Kok AH, de Waal MW, Husebo BS, Lautenbacher S, Kunz M, Scherder EJ, Corbett A. Pain management in patients with dementia. Clin Interv Aging. 2013;8:1471-82

Alzheimer's Disease International. Dementia Friendly Communities: key principles. 2016 Available at: https://www.alz.co.uk/adi/pdf/dfcprinciples.pdf (accessed October 31 2017)

Anand A, Khurana P, Chawla J, et al. Emerging treatments for the behavioral and psychological symptoms of dementia. CNS Spectr 2017; Sep 15:1-9. doi: 10.1017/S1092852917000530. [Epub ahead of print]

Apinis C, Tousignant M, Arcand M, et al. Can adding a standardized observational tool to interdisciplinary evaluation enhance the detection of pain in older adults with cognitive impairments? Pain Med 2014; 15: 32–41.

Australian Department of Health and Aged Care. Advocacy services for older people (website). Updated November 2016.

Australian Government. Federal Register of Legislation: User Rights Principles 2014. Available at https://www.legislation.gov.au/Details/F2014L00808 (accessed November 3rd 2017)

Azermai M. Dealing with behavioral and psychological symptoms of dementia: a general overview. Psychol Res Behav Manag 2015; 8: 181–85.

Bail K, Goss J, Draper B, Berry H, Karmel R, Gibson D. The cost of hospital-acquired complications for older people with and without dementia; a retrospective cohort study. BMC Health Serv Res 2015; 15: 91.

Bain KT, Schwartz EJ, Chan-Ting R. Reducing Off-Label Antipsychotic Use in Older Community-Dwelling Adults With Dementia: A Narrative Review J Am Osteopath Assoc 2017; 117:441–50.

Bensamoun D, et al Associations between Neuropsychiatric Symptoms and Cerebral Amyloid Deposition in Cognitively Impaired Elderly People. J Alzheimers Dis 2016; 49: 387–98.

Borson S, Frank L, Bayley PJ et al. Improving dementia care: the role of screening and detection of cognitive impairment. Alzheimers Dement 2013; 9: 151–9.

Burton CD, Entwistle VA, Elliott AM, et al. The value of different aspects of person-centred care: a series of discrete choice experiments in people with long-term conditions. BMJ Open 2017; 7:e015689.

Cousins JM, Bereznicki LR, Cooling NB, Peterson GM. Prescribing of psychotropic medication for nursing home residents with dementia: a general practitioner survey. Clin Interv Aging 2017; 12: 1573-78.

Cowdell F. The care of older people with dementia in acute hospitals. Int J Older People Nurs 2010; 5: 83–92

Deeks LS, Cooper GM, Draper B, et al. Dementia, medication and transitions of care. Res Social Adm Pharm 2016;12: 450–60.

Devshi R, Shaw S, Elliott-King J, et al. Prevalence of behavioural and psychological symptoms of dementia in individuals with learning disabilities. Diagnostic 2015; 5: 564–76.

Feast A, Moniz-Cook E, Stoner C, et al. A systematic review of the relationship between behavioral and psychological symptoms (BPSD) and caregiver well-being. International Psychogeriatrics 2016; 28: 1761–74.

Ferreira AR, et al. Needs in Nursing Homes and Their Relation with Cognitive and Functional Decline, Behavioral and Psychological Symptoms. Front Aging Neurosci 2016; 8: 72.

Ferreira, AR, et al. Behavioral and psychological symptoms: A contribution for their understanding based on the unmet needs model, In European Psychiatry, 2017; 41, (Suppl): S657.

Foebel AD, Liperoti R, Onder G, et al. Use of antipsychotic drugs among residents with dementia in European long-term care facilities: results from the SHELTER study. J Am Med Dir Assoc 2014; 15: 911–17.

Guideline Adaptation Committee. Clinical Practice Guidelines and Principles of Care for People with Dementia. Sydney. Guideline Adaptation Committee; 2016.

Haralambous B, Mackell P, Lin X, et al. Improving health literacy about dementia among older Chinese and Vietnamese Australians. Aust Health Rev 2017 Sep 7. doi: 10.1071/AH17056. [Epub ahead of print]

Herzig SJ, LaSalvia MT, Naidus E, et al. Antipsychotics and the Risk of Aspiration Pneumonia in Individuals Hospitalized for Nonpsychiatric Conditions: A Cohort Study. J Am Geriatr Soc 2017; Nov 2. doi: 10.1111/jgs.15066. [Epub ahead of print]

Hodgson NA, Gitlin LN, Winter L, Czekanski K. Undiagnosed illness and neuropsychiatric behaviors in community residing older adults with dementia. Alzheimer Dis Assoc Disord 2011; 25:109–15.

Huey ED, Lee S, Devanand DP. Brain regions involved in arousal and reward processing are associated with apathy in Alzheimer's disease and frontotemporal dementia. J Alzheimer Dis 2016; 55: 551–58.

Husebo BS, Ballard C, Cohen-Mansfield J, Seifert R, Aarsland D. The response of agitated behavior to pain management in persons with dementia. Am J Geriatr Psychiatry. 2014 Jul;22(7):708-17.

Kales HC, Gitlin LN, Lyketsos CG. Management of neuropsychiatric symptoms of dementia in clinical settings: recommendations from a multidisciplinary expert panel. Similar to DICE (diagnose, investigate, create, evaluate). J Am Geriatr Soc 2014; 62: 762–69.

Kleijer BC, van Marum RJ, Egberts AC, et al. The course of behavioral problems in elderly nursing home patients with dementia when treated with antipsychotics. Int Psychogeriatr. 2009; 21: 931–40.

Legere LE, et al. Non-pharmacological approaches for behavioural and psychological symptoms of dementia in older adults: A systematic review of reviews. J Clin Nurs 2017; Aug 9. doi: 10.1111/jocn.14007. [Epub ahead of print]

Lin S-Y. 'Dementia-friendly communities' and being dementia friendly in healthcare settings. Curr Opin Psychiatry 2017; 30: 145–50.

LoGiudice D. The health of older Aboriginal and Torres Strait Islander peoples. Australas J Ageing 2016;35: 82–85.

Loi SM, Westphal A, Ames D, et al. Minimising psychotropic use for behavioural disturbance in residential aged care. Aust Fam Physician 2015; 44: 180–84.

Long EM. An Innovative Approach to Managing Behavioral and Psychological Dementia. J Nurse Pract 2017; 13: 475–81.

Macfarlane S, Cunningham C. The need for holistic management of behavioral disturbances in dementia International Psychogeriatrics 2017; 29: 1055–58.

Makovac E, et al. Different Patterns of Correlation between Grey and White Matter Integrity Account for Behavioral and Psychological Symptoms in Alzheimer's Disease. J Alzheimers Dis. 2016; 50: 591-604.

Malara A, De Biase GA, Bettarini F, et al. Pain Assessment in Elderly with Behavioral and Psychological Symptoms of Dementia. J Alzheimers Dis 2016; 50: 1217–25.

McGovern J. The forgotten: Dementia and the aging LGBT community. Journal of Gerontological Social Work 2014; 57: 845–57.

McParland J, Camic PM. (a) Psychosocial factors and ageing in older lesbian, gay and bisexual people: A systematic review of the literature. J Clin Nurs 2016; Advanced publication. doi: 10.1111/jocn.13251.

McParland J, Camic PM. (b) How do lesbian and gay people experience dementia? Dementia 2016; May 9. pii: 1471301216648471. [Epub ahead of print]

McStay J. Advice on security of tenure. Australian Ageing Agenda 2014; Available at https://australianageingagenda.com.au/2014/02/13/advice-security-tenure/ (accessed November 2nd 2017)

Moreno A, Laoch A, Zasler ND. Changing the culture of neurodisability through language and sensitivity of providers: Creating a safe place for LGBTQIA+ people. NeuroRehabilitation. 2017; 41: 375–93.

Mukadam N, Sampson EL. A systematic review of the prevalence, associations and outcomes of dementia in older general hospital inpatients. Int Psychogeriatr. 2011 Apr;23(3):344-55.

Naqvi RM, Haider S, Tomlinson G, Alibhai S. Cognitive assessments in multicultural populations using the Rowland Universal Dementia Assessment Scale: a systematic review and meta-analysis. CMAJ 2015; 187(5): E169–75.

Nichols P, Horner B, Fyfe K. Understanding and improving communication processes in an increasingly multicultural aged care workforce. J Aging Stud. 2015 ;32: 23–31.

O'Neill N, Peisah C. Capacity and the Law. Updated June 2020. Available at austlii.community/wiki/Books/CapacityAndTheLaw/ (accessed July 15th 2021)

Peel E, Taylor H, Harding R. Sociolegal and practice implications of caring for LGBT people with dementia. Nurs Older People 2016; 28: 26–30.

Peisah C & Skladzien E. (2014) The use of restraints and psychotropic medications in people with dementia Paper 38: A report for Alzheimer's Australia. Alzheimer's Australia. www.dementia.org.au/sites/default/files/Publication_38_A4_print_version_Web.pdf Access 14th July 2021.

Perez Romero A, Gonzalez Garrido S. The importance of behavioral and psychological symptoms in Alzheimer's disease. Neurologia. 2016; DOI: 10.1016/j.nrl.2016.02.024.

Pinkert C, Faul E, Saxer S, et al. Experiences of nurses with the care of patients with dementia in acute hospitals: A secondary analysis. J Clin Nurs2017 Apr 20. doi: 10.1111/jocn.13864. [Epub ahead of print]

Price E. Pride or prejudice? Gay men, lesbians and dementia. Br J Social Work 2008; 38: 1337–52.

Radford K, Mack HA, Draper B et al. Prevalence of dementia in urban and regional Aboriginal Australians. Alzheimer's & Dementia 2015; 11: 271–79.

Saini G, Sampson EL, Davis S, et al. An ethnographic study of strategies to support discussions with family members on end-of-life care for people with advanced dementia in nursing homes. BMC Palliat Care 2016; 15: 55.

Sampson EL, Leurent B, Blanchard MR, Jones L, King M. Survival of people with dementia after unplanned acute hospital admission: a prospective cohort study. Int J Geriatr Psychiatry 2013; 28: 1015–22.

Savaskan E, Bopp-Kistler I, Buerge M, et al. Therapy guidelines for the behavioural and psychological symptoms of dementia. Praxis 2014; 103: 135–48.

Shin H-Y, Gadzhanova S, Roughead EE, et al. The use of antipsychotics among people treated with medications for dementia in residential aged care facilities. International Psychogeriatrics 2016; 28: 977–82.

Smith K, Flicker L, Atkinson D, et al. The KICA Carer: informant information to enhance the Kimberley Indigenous Cognitive Assessment. Int Psychogeriatr 2016; 28: 101–7.

Smith K, Flicker L, Lautenschlager NT et al. High prevalence of dementia and cognitive impairment in Indigenous Australians. Neurology 2008; 71: 1470–73.

Smith SK, Dixon A, Trevena L, et al. Exploring patient involvement in healthcare decision making across different education and functional health literacy groups. Social Sci Med 2009; 69: 1805–12.

Steinberg M, Shao H, Zandi P, et al. Point and 5-year period prevalence of neuropsychiatric symptoms in dementia: the Cache County Study. Int J Geriatr Psychiatry 2008; 23: 170–77.

Stewart J, Lohoar S, Higgins D. Effective Practices for Service Delivery Coordination in Indigenous Communities. Canberra: Australian Institute of Health and Welfare, 2011. Resource Sheet No. 8. Available at: http://www.aboriginal-affairs.nsw.gov.au/pdfs/about/closing-the-gap.pdf (accessed November 3rd 2017)

Stratton RJ, King CL, Stroud MA, Jackson AA, Elia M. 'Malnutrition Universal Screening Tool' predicts mortality and length of hospital stay in acutely ill elderly. Br J Nutr 2006; 95: 325–30.

Sutin AR, Stephan Y, Luchetti M, et al. Self-reported personality traits are prospectively associated with proxy-reported behavioral and psychological symptoms of dementia at the end of life. Int J Geriatr Psychiatry 2017 Sep 4. doi: 10.1002/gps.4782. [Epub ahead of print]

Tanaka T. Factors predicting perioperative delirium and acute exacerbation of behavioral and psychological symptoms of dementia based on admission data in elderly patients with proximal femoral fracture: A retrospective study. Geriatr Gerontol Int 2016; 16: 821-28.

Therapeutic Goods Administration. Medicines Safety Update August 2015. Available at: https://www.tga.gov.au/publication-issue/medicines-safety-update-volume-6-number-4-august-2015 (Accessed November 4th 2017)

Tible OP, Riese F, Savaskan E, et al. Best practice in the management of behavioural and psychological symptoms of dementia. Ther Adv Neurol Disord 2017;10: 297–309.

Tjia J, Lemay CA, Bonner A3, et al. Informed Family Member Involvement to Improve the Quality of Dementia Care in Nursing Homes. J Am Geriatr Soc. 2017; 65: 59-65.

Travers C, Byrne GJ, Pachana NA, et al. Prospective observational study of dementia in older patients admitted to acute hospitals. Australas J Ageing 2014; 33: 55–58.

van der Linde RM, et al. Longitudinal course of behavioural and psychological symptoms of dementia: systematic review. Br J Psychiatry 2016; 209: 366–77.

Walsh KA, Dennehy R, Sinnott C, et al. Influences on Decision-Making Regarding Antipsychotic Prescribing in Nursing Home Residents With Dementia: A Systematic Review and Synthesis of Qualitative Evidence. J Am Med Dir Assoc 2017; 18: 897.e1-897.e12. doi: 10.1016/j.jamda.2017.06.032. Epub 2017 Aug 12.

White N, Leurent B, Lord K, et al. The management of behavioural and psychological symptoms of dementia in the acute general medical hospital: a longitudinal cohort study. Int J Geriatr Psychiatry 2017; 32: 297–305.

Zdanys KF, Carvalho AF, Tampi RR, Steffens DC. The Treatment of Behavioral and Psychological Symptoms of Dementia: Weighing Benefits and Risks. Curr Alzheimer Res 2016; 13: 1124–33.

Zhao Q-F, Tan L, Wang H-F, et al. The prevalence of neuropsychiatric symptoms in Alzheimer's disease. Systematic review and meta-analysis. J Affective Disord 2016; 190: 264–71.
